# Multiple interacting environmental drivers reduce the impact of solar UVR on primary productivity in Mediterranean lakes

**DOI:** 10.1038/s41598-020-76237-5

**Published:** 2020-11-13

**Authors:** Marco J. Cabrerizo, E. Walter Helbling, Virginia E. Villafañe, Juan M. Medina-Sánchez, Presentación Carrillo

**Affiliations:** 1 Departamento de Ecología y Biología Animal, Facultad de Ciencias del Mar, Universidad de Vigo, Campus Lagoas Marcosende, s/n, 36310 Vigo, Spain; 2 Centro de Investigación Mariña da Universidade de Vigo (CIM-UVigo), Illa de Toralla s/n, 36331 Vigo, Spain; 3 Estación de Fotobiología Playa Unión, Casilla de Correos 15, 9103 Rawson, Chubut Argentina; 4 Consejo Nacional de Investigaciones Científicas y Técnicas (CONICET), Buenos Aires, Argentina; 5 Departamento de Ecología, Facultad de Ciencias, Universidad de Granada, Campus Fuentenueva s/n, 18071 Granada, Spain; 6 Instituto Universitario de Investigación del Agua, Universidad de Granada, C/Ramón Y Cajal, no. 4, 18071 Granada, Spain

**Keywords:** Climate-change ecology, Ecophysiology, Freshwater ecology, Ecology, Limnology

## Abstract

Increases in rainfall, continental runoff, and atmospheric dust deposition are reducing water transparency in lakes worldwide (i.e. higher attenuation *Kd*). Also, ongoing alterations in multiple environmental drivers due to global change are unpredictably impacting phytoplankton responses and lakes functioning. Although both issues demand urgent research, it remains untested how the interplay between *Kd* and multiple interacting drivers affect primary productivity (P^c^). We manipulated four environmental drivers in an in situ experiment—quality of solar ultraviolet radiation (UVR), nutrient concentration (Nut), CO_2_ partial pressure (CO_2_), and light regime (Mix)—to determine how the P^c^ of nine freshwater phytoplankton communities, found along a *Kd* gradient in Mediterranean ecosystems, changed as the number of interacting drivers increased. Our findings indicated that UVR was the dominant driver, its effect being between 3–60 times stronger, on average, than that of any other driver tested. Also, UVR had the largest difference in driver magnitude of all the treatments tested. A future UVR × CO_2_ × Mix × Nut scenario exerted a more inhibitory effect on P^c^ as the water column became darker. However, the magnitude of this synergistic effect was 40–60% lower than that exerted by double and triple interactions and by UVR acting independently. These results illustrate that although future global-change conditions could reduce P^c^ in Mediterranean lakes, multiple interacting drivers can temper the impact of a severely detrimental driver (i.e. UVR), particularly as the water column darkens.

## Introduction

Solar radiation, including the ultraviolet (UVR, 280–400 nm), constitutes the main energy source for aquatic autotrophic microorganisms^1^, especially phytoplankton. In aquatic habitats, the attenuation of solar radiation depends on several factors: the water itself; the presence of chromophoric dissolved organic (DOM) and inorganic matter; the concentration of organic and inorganic particles; and the density of phytoplankton which can act as a self-shading agent^2^. Because these factors alter the intensity, and spectral composition of the underwater light environment, they can be considered key modulators of the phytoplankton responses to UVR.

Together with solar UVR, three other major drivers alter phytoplankton community responses: (1) nutrient (Nut) concentration in surface waters due to more continental runoff and/or atmospheric dust deposition, as currently being registered in tropical, temperate, and polar lakes^3,4^, (2) increasing concentrations of atmospheric carbon dioxide (pCO_2_) derived from burning of fossil fuels by humans^5^, and (3) recurrent changes in mixing conditions (Mix) due to the increasing in the frequency and intensity of extreme events (e.g. high winds, precipitation associated with storms)^6^.

Many studies related to global-change impact have focused on the individual effects of drivers on specific environments and conditions^7,8^. Recent reports propose that quantifying interactions including several drivers are essential because the effects usually do not follow the same direction^9–11^. For example, Nut inputs reportedly stimulated phytoplankton biomass, but greater CO_2_ concentrations exerted no significant effect on this variable; however, when both drivers acted together they synergistically boosted C biomass^12,13^. Also, Carrillo et al*.*^14^ reported no effect of UVR on phytoplankton biomass growth or primary production (PP); however, Nut-enrichment unmasked a damaging UVR effect, inhibiting both processes. Furthermore, the net effects of opposing drivers are species specific^15^, depending on an organism’s capability to overcome environmental stress, as well as context dependent^16^. In this sense, Carrillo et al*.*^17^ found a synergistic UVR × Nut effect that stimulated PP and biomass in a highly transparent Mediterranean lake, but an antagonistic effect in a less transparent Andean lake. Likewise, the interaction between UVR and fluctuating light regimes (Mix) also exerted a synergistic effect by increasing the inhibition of PP and boosting the excretion of organic carbon (EOC) in a turbid lake, whereas the opposite occurred in clear lakes, with Mix counteracting the harmful UVR effects^18^.

Lakes can be considered reference ecosystems for assessing the repercussions of global change^19^. These ecosystems have diverse biota, register the highest productivity per unit area, and sequester C at rates roughly one order of magnitude greater than any other ecosystem^20,21^. However, they are the most vulnerable ecosystems due to recurrent shifts in the light regime (from clear to turbid waters) derived from global climate change and human activities^22^. Despite the ecological relevance of lakes for the C-cycle, no in situ experimental studies have simultaneously tested how the effects of UVR on natural phytoplankton communities could be altered when interacting with CO_2_, Mix, and Nut. Additionally, no study has quantified the extent to which such a multi-driver scenario can be modulated by shifts in the underwater light environment. Indeed, Hilt et al*.*^22^ recently called attention to the need for experimental studies on the effects of light-regime shifts in a global-change context. Currently, only 4% of the studies published on this topic have quantified, on an observational basis, how changes in the light regime alter key ecosystem functions (e.g. PP). Also, previous results reported by Helbling et al*.*^23^ have demonstrated that the interaction between UVR and Mix lowered PP with the darkening of the underwater environment due to increasing DOM concentrations. In addition, other studies have showed that DOM exerts a sheltering effect by stimulating the growth of nanoplanktonic flagellates and diatoms in comparison to the growth of microplankton^24,25^. Thus, the acclimation capacity/time of phytoplankton in clear vs. dark environments proves crucial in these situations. DOM can also stimulate the microbial loop, by providing a nutrient subsidy to bacteria, and by reducing the exposure of protists to damaging UVR^26^.

Thus, in short, it can be considered that: (1) UVR is a potentially harmful driver of communities^27^; (2) UVR harmfulness increases with the darkening of the environment because their communities are adapted to low-light conditions, and thus they have weaker photoprotective mechanisms (e.g. fewer micosporine-like amino acids) than do cells acclimated to high-light conditions^23,28,29^; and (3) biotic responses to multiple drivers, at the population level, depend on the response to the single dominant driver^9^. On this basis, we hypothesised that increases in CO_2_, and Nut under fluctuating light regimes (Mix) will accentuate the inhibitory effect of UVR on primary productivity (P^c^), and that such effect will be stronger in communities inhabiting darker environments. To test our hypothesis, we used phytoplankton communities from nine Mediterranean lakes with different underwater radiation environments (i.e. different attenuation coefficients, as for example, *Kd*_PAR_), exposed them in situ to a complex multi-driver scenario (UVR × CO_2_ × Mix × Nut), and measured the P^c^ under these conditions, using a full factorial approach.

## Results

### Water transparency of Mediterranean lakes in worldwide comparison

**Figure 1:**
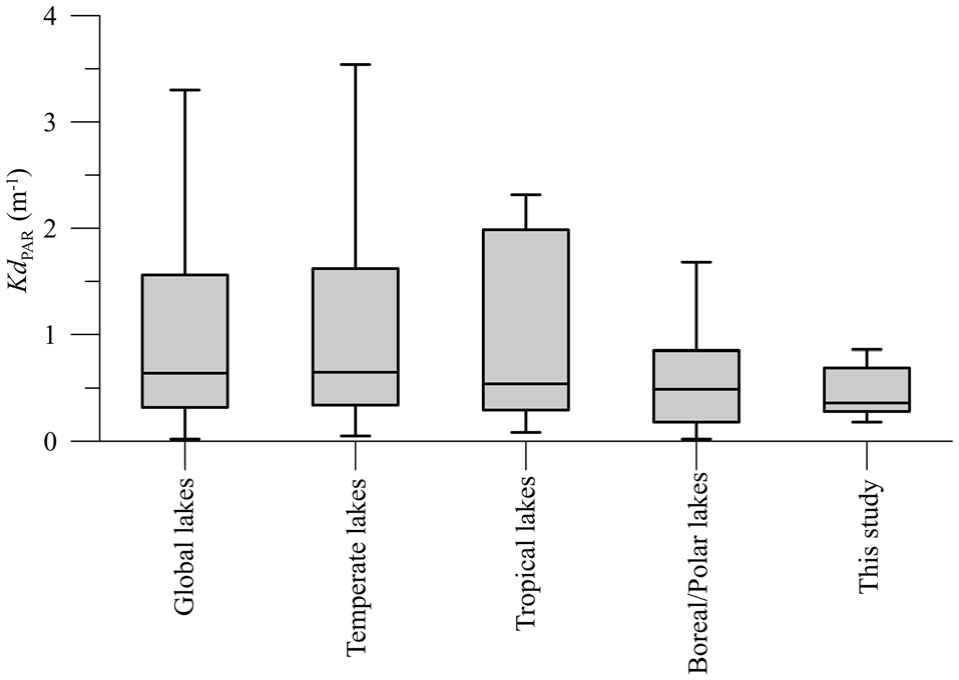
Box plots showing the distribution of the photosynthetically active radiation attenuation coefficient (as *Kd*_PAR_, m^−1^) for global lakes (n = 421); the lakes were sorted by climatic areas (temperate, tropical, boreal, and polar), and compared with the lakes where the experiments were performed. The boxes show the median *Kd*_PAR_ plus the lower (25%) and upper (75%) quartile while the whiskers indicate 1.5-times the interquartile range.

We used the attenuation of PAR (400–700 nm) as a measure of transparency of the water column; we found low median values of *Kd*_PAR_ in aquatic ecosystems worldwide (~ 0.50 m^−1^), implying generally high transparency at these wavelengths of solar radiation (Fig. 1). Median *Kd*_PAR_ values were 0.49 m^−1^ in boreal/polar, to 0.65 m^−1^ in temperate, and 0.54 m^−1^ in tropical lakes. No significant differences in *Kd*_PAR_ were found among climatic areas due to its high variability (LSD post hoc test, p > 0.20; n = 421). In our experiments with phytoplankton communities from nine Mediterranean lakes, the water had a *Kd*_PAR_ gradient of 0.18–0.90 m^−1^, this falling within the range found on a global scale (Fig. 1).

### Physico-chemical and biological conditions in Mediterranean lakes

**Figure 2:**
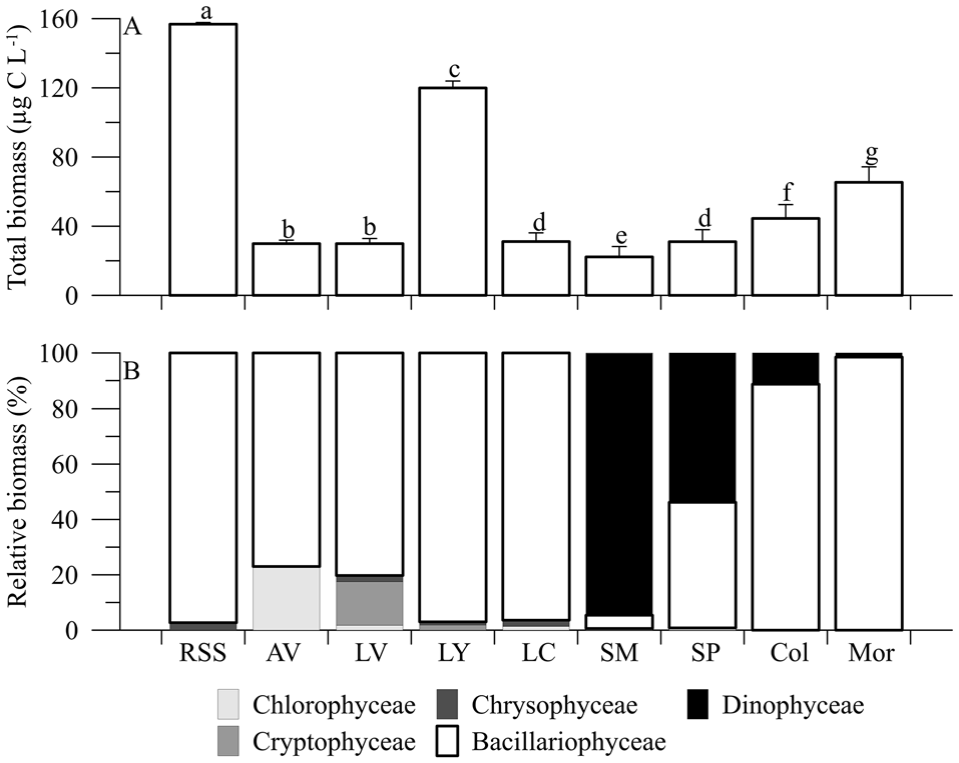
Relative biomass (%) of phytoplankton communities from Lakes Río Seco Superior (RSS), Aguas Verdes (AV), Lagunillos de la Virgen (LV), Las Yeguas (LY), La Caldera (LC), Santos Morcillo (SM), San Pedro (SP), Colgada (CO) and Morenilla (MO).

Water-column temperatures ranged from ~ 10 to 23 °C, and the total biomass ~ 40 and 160 µg C L^−1^ (Table 1). Chlorophyll *a* (Chl *a*) and nutrient concentrations indicated oligo-mesotrophy in the lakes sampled, i.e. < 6 µg L^−1^ for Chl *a*, and < 0.50, < 10, < 620, < 230 µM for total phosphorus (TP), silicate (Si), total nitrogen (TN), and dissolved organic carbon (DOC), respectively (Table 1). The bulk of phytoplankton biomass consisted of diatoms (mainly *Cyclotella* sp.), except for the lakes San Pedro (SP) and Santos Morcillo (SM), where dinoflagellates (mainly *Peridinium* sp.) accounted for most of the biomass (Fig. 2).

**Table 1:** Location (latitude/longitude), height, water transparency (as *Kd*) (for UVR i.e., 305, 320 and 380 nm and photosynthetically active radiation [PAR]), temperature (T) and mean concentrations (± SD) of total phosphorus (TP) and nitrogen (TN), silicate (SiO_3_^2−^), dissolved organic carbon (DOC), chlorophyll *a* (Chl *a*) concentrations, and total biomass in lakes Río Seco Superior (RSS), Aguas Verdes (AV), Lagunillo Grande de la Virgen (LV), Las Yeguas (LY) and La Caldera (LC), Santos Morcillo (SM), San Pedro (SP), Colgada (CO) and Morenilla (MO).

Variable	RSS	AV	LV	LY	LC	SM	SP	CO	MO
Location (lat/long)	37° 03′ N	37° 02′ N	37° 03′ N	37° 03′ N	37° 03′ N	38° 57′ N	38° 55′ N	38° 57′ N	38° 59′ N
	3° 20′ W	3° 22′ W	3° 22′ W	3° 22′ W	3° 19′ W	2° 51′ W	2° 50′ W	2° 52′ W	2° 53′ W
Height (m.a.s.l.)	3052	3050	2950	2880	3050	803	832	790	763
*Kd*_305_ (m^−1^)	4.46	2.06	1.13	0.5	0.44	0.3	0.86	1.22	0.99
*Kd*_320_ (m^−1^)	3.82	1.83	1.03	0.40	0.37	0.36	0.93	2.08	0.74
*Kd*_380_ (m^−1^)	2.16	1.04	0.74	0.21	0.26	0.27	0.79	0.53	0.50
*Kd*_PAR_ (m^−1^)	0.86	0.69	0.72	0.18	0.29	0.20	0.41	0.28	0.36
T (°C)	17.9	19.5	9.8	14.28	11.76	23.43	22.09	22.05	22.90
TP (µM)	0.74 (0.08)	0.36 (0.00)	0.17 (0.01)	0.15 (0.01)	0.34 (0.12)	0.29 (0.06)	0.47 (0.10)	0.17 (0.02)	0.31 (0.02)
TN (µM)	19.28 (0.71)	16.43 (5.71)	11.43 (1.42)	15.71 (2.14)	10.71 (0.71)	575.71 (35.72)	619.30 (35.71)	578.57 (28.58)	512.86 (34.29)
SiO_3_^2−^ (µM)	1.79 (0.00)	2.50 (0.00)	3.21 (0.00)	1.78 (0.00)	1.79 (0.36)	7.14 (0.00)	6.10 (0.36)	9.30 (0.35)	8.93 (0.71)
DOC (µM)	166.67 (29.17)	232.50 (10.83)	75.83 (5.00)	73.33 (49.17)	123.33 (6.67)	200 (47.50)	226.67 (78.33)	136.67 (25.00)	137.50 (8.33)
Chl *a* (µg L^−1^)	2.69 (0.23)	1.34 (0.17)	1.40 (0.25)	2.07 (0.31)	3.48 (0.39)	2.04 (0.49)	5.50 (0.29)	1.71 (0.32)	3.17 (1.98)
Biomass (µg C L^−1^)	156.77 (2.12)	29.90 (2.06)	29.92 (1.12)	119.92 (1.41)	31.13 (2.33)	22.23 (1.33)	31.02 (2.89)	44.49 (1.99)	65.35 (4.84)

### Individual and interactive effects of UVR, CO_2_, Mix and Nut on P^c^

**Figure 3:**
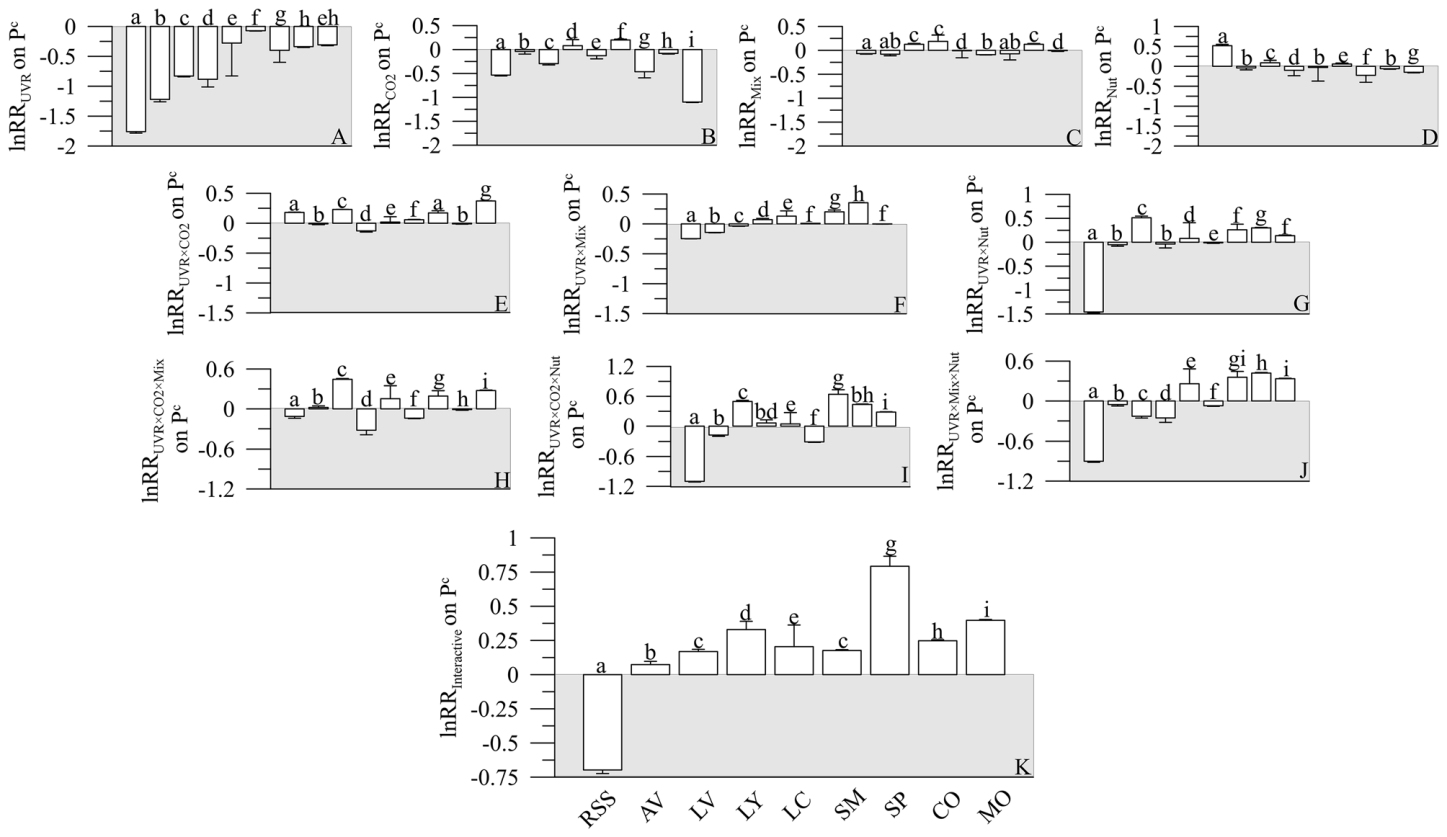
Natural logarithm response ratios (lnRR) for the individual effect of UVR (**A**), CO_2_ (**B**), mixing—Mix (**C**) and nutrients—Nut (**D**), their 2- (**E**–**G**), 3- (**H**–**J**) and 4-level lnRR_Int_ (**K**) interaction in Lakes Río Seco Superior (RSS), Aguas Verdes (AV), Lagunillos de la Virgen (LV), Las Yeguas (LY), La Caldera (LC), Santos Morcillo (SM), San Pedro (SP), Colgada (CO) and Morenilla (MO). The bars represent the mean of three replicates, and the vertical lines the pooled standard deviation for equal sample size (see “Methods”). The letters on top of bars indicate significant differences by the Least Significant Differences (LSD) post hoc test. Note different y-axis scales. The values > 0 denote a synergistic effect, and < 0 an antagonistic effect.

The P^c^ rates ranged between 0.02 and 0.03 and 0.80 h^−1^ (Fig. S1). In six of the nine lakes sampled, all drivers tested decreased the P^c^ (ranging − 0.05 and − 1.80) although Nut and Mix proved less inhibitory [ln response ratio (lnRR) < 0.52 in all cases] than did UVR or CO_2_ (Fig. 3A–D).

UVR was the dominant driver because the magnitude of its effect was higher (ca. 3–60-fold in average) than that exerted individually by all the other drivers tested. Also, UVR had the largest difference in driver magnitude of all treatments. Double and triple interactions among drivers revealed two response patterns: (1) a weaker inhibitory effect exerted by each single driver on P^c^, particularly UVR and CO_2_; and (2) an antagonistic effect on P^c^ in ~ 50% of the double and triple interactions tested (Fig. 3E–J). The UVR × CO_2_ × Nut × Mix interaction synergistically reduced P^c^ (i.e. Río Seco Superior; Fig. 3K). However, this negative effect (minimum lnRRinteractive values < − 0.7) was some 40–60% lower than exerted in 2-level (i.e. minimum lnRR values of ~ − 1.5), and 3-level (minimum lnRR values of ~ − 1.2) interactions and when UVR acted alone (minimum lnRR values ~ − 2). Finally, we found no significant effect for the TN:TP ratios, in situ temperature, or mean solar irradiance during exposure on the P^c^ response to UVR, CO_2_, Mix, and Nut, and the interaction of any of these factors (Table S2).

### Relationship between shifts in the ***Kd***_PAR_ and P^c^

**Figure 4:**
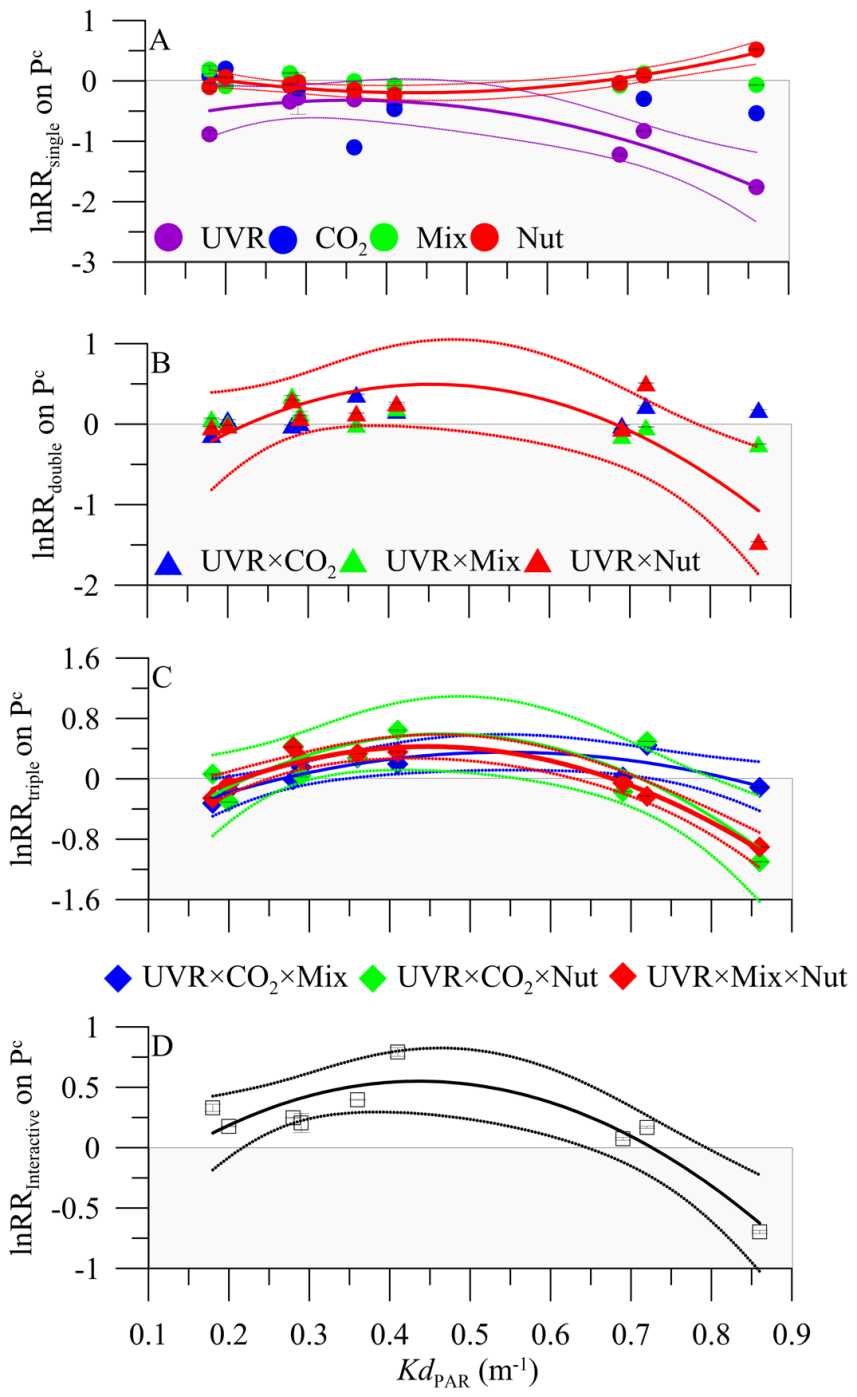
Natural logarithm response ratios (lnRR) of the single and interactive effects of ultraviolet radiation (UVR), carbon dioxide (CO_2_), mixing (Mix) and nutrients (Nut) as a function of the photosynthetically active radiation attenuation coefficient (*Kd*_PAR_, m^−1^) for the lakes considered in the study. The bars represent the mean of three replicates, and the vertical lines the pooled standard deviation for equal sample size (see “Methods”). The solid and dashed lines represent the fitted polynomial regression lines and the 95% confidence bands, respectively. The values > 0 denote a synergistic effect, and < 0 an antagonistic effect.

From our experimental phytoplankton communities, which were acclimated to different light environments, Fig. 4 presents the results for the individual and interactive lnRR on P^c^ as a function of the *Kd*_PAR_ gradient. We found that UVR and Nut had an increasing or decreasing inhibitory effect, respectively, on P^c^ with *Kd*_PAR_, reaching a maximum at ~ 0.90 m^−1^. By contrast, CO_2_ and Mix had no significant effect on P^c^ along the *Kd*_PAR_ gradient (Fig. 4A; Table S3). Evaluating the double and triple interactions, we found a unimodal response pattern, with a significant shift from a slight antagonism to a synergism on P^c^ with increasing *Kd*_PAR_ under the combinations UVR × Nut (Fig. 4B; Table S3), UVR × CO_2_ × Mix, UVR × CO_2_ × Nut and UVR × Mix × Nut (Fig. 4B,C; Table S3). Similarly, the lnRR_Interactive_ showed a similar unimodal response, i.e. from an antagonistic [+ 0.75] to a synergistic effect [− 0.70] over the *Kd*_PAR_ gradient (Fig. 4D; Table S3). Altogether, the mean lnRR_single_ was − 0.23, while lnRR_double_ and lnRR_triple_ were 0.04 and 0.03, respectively, and lnRR_interactive_ was 0.19. This signifies that the inhibitory effect (mostly by UVR) decreased as the number of interacting drivers increased.

## Discussion

Our work evidences that water transparency (i.e. estimated as K*d*_PAR_) can be a key predictor of the effects of multiple environmental drivers may exert on P^c^ in Mediterranean lakes. Although our findings do not allow global projections of trends (because they were focused on Mediterranean lakes), here we show that an increasing environmental complexity reduces the magnitude of the individual effects of different global-change drivers, particularly those of UVR, the dominant driver. These findings at the community level extend the proposal by Brennan and Collins^9^ who used a model green algal species to state that the greater number of interacting drivers, the more likely that the interaction contains at least one severely detrimental driver, and therefore, that the biotic response to multiple environmental drivers greatly depends on the response to the single dominant driver (i.e. UVR in our case).

It bears mentioning that these results represent acute responses of phytoplankton physiology to multiple interacting drivers because our short-term in situ experiments did not let these communities to acclimate/adapt to the predicted environmental conditions. Still, our results are quite realistic for the following reasons: (1) we exposed the communities to predicted future environmental scenarios (RCP 8.5 scenario)^30^, and (2) we worked with natural communities already adapted to different in situ environmental conditions that resembled the median *Kd*_PAR_ values found in freshwater ecosystems worldwide (Fig. 1).

We found that UVR was the driver that exerted the strongest synergistic (and inhibitory) effect on P^c^, intensifying with the darkening of the water column. These findings are consistent with the results of Helbling et al*.*^18,23^, who reported a maximum UVR inhibitory effect on P^c^ in turbid rather than clear environments. This response pattern could be explained by the fact that the dominant phytoplankton groups in our lakes (diatoms and dinoflagellates) have lower amounts of photoprotective compounds (e.g. mycosporine-like amino acids [MAAs]) as compared to other phytoplankton groups^31^. Since DOM also acts as a shelter for solar radiation, communities inhabiting these ecosystems have lower natural amounts of MAAs than do organisms adapted to clear (and highly UVR-exposed) environments^28,32^. The lower photoprotective capacity could in turn support the reduced P^c^ reported in our experiments. The potential mechanism underlying this response could involve alterations of the Calvin cycle and RuBisCO regulation^33^. This downregulation mechanism would cause the electron-transport system to accumulate excessive reducing power that could not be dissipated as heat through non-photochemical quenching (NPQ), thus ultimately depressing photosynthesis [e.g. UVR^18^; fluctuating light^34^]. Nevertheless, we speculate that the reduced P^c^ could result when a fraction of the C incorporated by photosynthesis is diverted to synthesize ATP and C-rich storage products (e.g. polysaccharides). These molecules are required for energetically costly processes (e.g. photorespiration, nutrient uptake, electron flows) that enable phytoplankton to cope with stressful environmental conditions^35^.

Partially in agreement with our hypothesis, the UVR × CO_2_ × Nut × Mix scenario exerted a synergistic effect on P^c^ at *Kd*_PAR_ > 0.7 m^−1^. However, contrary to our expectations, this synergistic effect in darker waters was of lower magnitude under UVR × CO_2_ × Mix × Nut than that caused by UVR × Nut, UVR × CO_2_ × Nut, UVR × Mix × Nut or by the single UVR effect. The mechanisms underlying this response pattern could be an increased productivity efficiency by natural phytoplankton communities, as a strategy to compensate for reduced photon fluxes in darker environments^36^. It is plausible that the chronic exposure, and consequently the adaptation already under way in communities naturally exposed to high UVR levels, had produced a stress-induced community tolerance^37^. Nevertheless, it should not being forgotten that the inherent greater environmental variability of smaller aquatic ecosystems (i.e. ponds, lakes) also fosters greater potential to cope with the impacts of multiple drivers^10^. As the effects of UVR, CO_2_, Mix, and Nut on phytoplankton vary according to its ecophysiological traits and the ecosystem properties, the way in which the intensity and duration of such drivers will impact aquatic ecosystems in a stressful world remains as a challenge for scientific community.

Within this framework, we suggest that communities from darker environments (e.g. humic lakes, deep epilimnion, estuarine areas) may have a potential competitive advantage when multiple drivers interact. Because ~ 60% of the total solar energy absorbed by phytoplankton in surface clear waters is dissipated as heat (i.e. as NPQ), phytoplankton inhabiting the most illuminated layer of aquatic ecosystems would be operating at about half of their maximal photosynthetic energy-conversion efficiency^38,39^. According to our findings, we could expect a reduction of the maximal photosynthetic efficiency in phytoplankton communities to be lower in darker than in clear surface waters of Mediterranean lakes under the action of multiple drivers. Although each ecosystem has its own particularities, we can rule out the possibility that our results were biased by interference derived from different nutrient ratios, in situ temperatures, or variable mean solar irradiance, as we found no significant effect of these three drivers on P^c^.

## Conclusion

Our research adds to the recent evidence indicating major changes in the structure of the planktonic communities when lakes undergo a light-regime shift towards turbid environments^40^. We propose that low water transparency under multiple interacting drivers may have a synergistic effect on near-surface P^c^, reducing it by 40%; however, the magnitude of such negative impact would be 40–60% lower than when UVR acts as a single driver. Because our results refer to small spatial and short temporal scales, further studies performed over longer-term scales and at the ecosystem level would enable planners not only to quantify the magnitude of global change with more accuracy but also to design more appropriate management and conservational strategies.

## Methods

### Literature review: water transparency in lakes

We surveyed the literature from 1960 to 2018 through Scopus using “*lake, ultraviolet radiation, attenuation, lake water, dissolved organic carbon, organic matter and freshwater environment*” as keywords, along with unpublished data sources by our group, and we found a total of 421 valid estimates related with *Kd*_PAR_ (“Supplementary dataset”). From this dataset, we calculated the median *Kd*_PAR_ for temperate, tropical, and boreal/polar lakes. We used *Kd*_PAR_ as a proxy of the underwater light environment under which phytoplankton is adapted because it is an inherent property of each water mass sampled and consequently does not depend on transient weather conditions such as incident solar radiation. In addition, *Kd*_PAR_ data are easily available in the literature in comparison with other better descriptors of the underwater light environment such as average irradiance.

## Experimental study

Nine lakes from the Sierra Nevada National Park and Lagunas de Ruidera Natural Park were used to establish a gradient of *Kd*_PAR_. Lakes from Sierra Nevada National Park are mixed, oligotrophic high-mountain lakes located above the tree line on a siliceous bedrock in a glacial cirque^41^, whereas lakes in Lagunas de Ruidera Natural Park are mixed, oligotrophic ecosystems with high nitrate concentrations from land use, located on calcareous substrates^42^. Phytoplankton communities from these lakes have been the focus of recent studies aiming to determine the single effects of solar UVR, or their interaction in 2-level combinations, such as nutrient inputs^14,42–44^, vertical mixing or stratification^18,45^ and/or increased temperatures^46,47^ on PP.

### Sampling and experimental setup

Surface-water samples (0.5 m depth) were collected on July 2012 from the lakes Río Seco Superior (RSS; day 8), La Caldera (LC; day 10), Aguas Verdes (AV; day 10), Las Yeguas (LY; day 12), Lagunillo Grande de la Virgen (LV; day 12), Santos Morcillo (SM; day 17), San Pedro (SP; day 19), Colgada (CO; day 20), and Morenilla (MO; day 21) using a 6-L acid washed (1 N HCl) Van Dorn bottle, pre-screened through a 45 µm Nitex mesh to remove the large zooplankton, and placed in 10-L acid-washed opaque containers (30 L in total), and transported in darkness to the closest laboratory (between 1 and 3 h away from the sampling sites). Despite the fact that all phytoplankton communities came from lakes with different physico-chemical and biological conditions (Table 1), we exposed them to the same experimental manipulation procedure and drivers, as described below.

**Figure 5:**
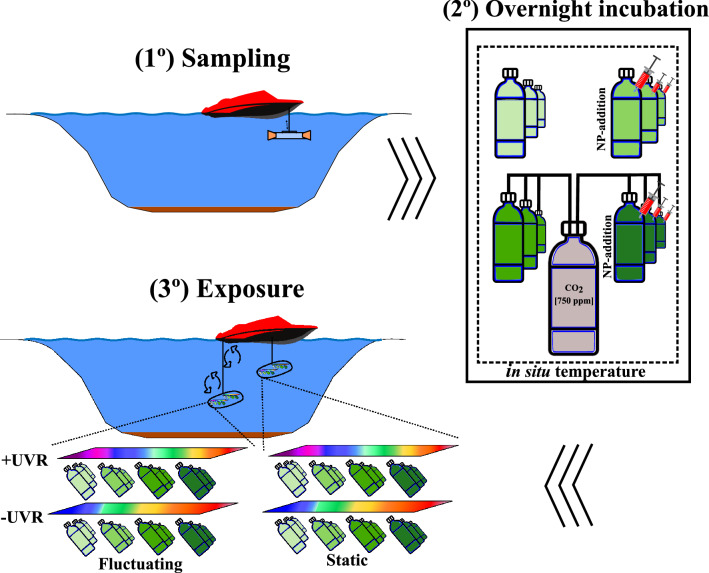
Graphic scheme of the experimental design: (1) sampling, in which phytoplankton communities were sampled; (2) overnight incubation, in which phytoplankton communities sampled were exposed overnight to both ambient and a nutrient pulse, under ambient and increased pCO_2_; and (3) exposure, in which phytoplankton communities incubated under the nutrients and pCO_2_ treatments mentioned were exposed in situ for 4 h centred on local noon to two solar radiation qualities: + UVR (> 280 nm) and − UVR (> 400 nm), and two light regimes: static (0.5 m depth) vs. fluctuating (moving up/down between 0 and 3 m depth).

Once in the laboratory, the original water sample collected from each lake (sampling—Fig. 5) was divided into 12 2-L polyethylene terephthalate (PET) bottles, maintained at the in situ temperature of the lake in a temperature-controlled room and incubated overnight under two pCO_2_ and two nutrient concentrations (incubation overnight—Fig. 5). The + CO_2_ level was maintained by constant bubbling throughout the night (12 h) from a gas tank at 750 ppm (Air Liquide, S.A.) to reach the pCO_2_ predicted under the RCP 4.5 scenario^30^ whereas the − CO_2_ treatment was simulated by constant air bubbling to the samples (same as above) using an air pump. The pH of the samples was measured before and after 12 h of bubbling using a potentiometric titrator (Titrando 905, Metrohm, USA, Inc.) equipped with the Tiamo titration software v 2.0. The total CO_2_ in the water samples was calculated from alkalinity and pH measurements^48^.

After the incubation period, subsamples coming from each 2-L-bottle and experimental treatment were subsequently placed into 50-mL quartz vessels, transported and incubated in the lakes for 4 h centred at local noon under two light qualities and two Mix treatments (exposure—Fig. 5).

The Mix treatments were applied by using a customized mixing simulator equipped with a frequency-controlled DC motor (Maxon motor, Switzerland) that maintains a constant velocity (1 m every 4 min, ten cycles in total) throughout the incubations. Overall, all samples incubated at 0.5 m depth received mean irradiance values comparable to those of samples moving in the upper 3 m of the water column during the exposure period (UVR *t* test = 1.68, p = 0.17 ; PAR *t* test = 1.96, p = 0.12; mean values Table S1), although lake-specific differences existed. Thus, and due to the differential attenuation of solar radiation in the lakes tested, it was not possible to completely match both UVR and PAR in all lakes (Table S1). Both trays were placed ~ 2 m from the side of the boat, using an aluminium pole so that they were not shaded by the boat and had no interference from the shoreline during the incubation period.

A 2 × 2 × 2 × 2 full factorial design (in triplicate) was implemented for each lake with the following factors:

1. the **UVR** factor, with two qualities of solar radiation: − UVR (samples receiving only PAR, > 400 nm) with the quartz vessels covered with UVR-filter foil (UV-Process Supply Inc., IL, USA) and, + UVR (samples receiving UVR + PAR, > 280 nm) with uncovered quartz vessels. This treatment is intended for the evaluation of the net UVR effect because the experimental lakes are exposed to extreme UVR levels during spring–summer days (noon irradiances: ~ 6/30/70 µW cm^−2^ for 305/320/380 nm, respectively).
2. The **CO**_**2**_ factor, with two pCO_2_ levels: − CO_2_ (400 ppm) and + CO_2_ (750 ppm). The pH values (decreased by ~ 0.28 units), and pCO_2_ mimics those predicted by the IPCC^30^ by 2100 (RCP8.5 scenario).
3. The **Mix** factor, with two light regime levels, static (Stat), with samples placed at a fixed depth (0.5 m), and fluctuating (Fluc), with samples moving up and down from surface to 3 m deep (see above). The mixing speed imposed to the samples was maintained the same in all lakes (see above), and it resembled the mean velocities measured in situ during the sampling day. For this, we measured the effective quantum yield of the communities (i.e. a proxy of the photosynthetic activity) at the surface and at different depths in the water column at noon. From these values, by applying the model presented in Villafañe et al*.*^49^, we determined the phytoplankton mixing speed in the water column over short-term scales. The rationale behind using the same mixing speed was to expose all the communities to the same experimental manipulation, as we did with other drivers tested.
4. The **Nut** factor, with two nutrient levels: Amb, with nutrient concentrations that were not manipulated, and Enr, through the addition of 1 µM P (as NaH_2_PO_4_). 29.03 µM of inorganic nitrogen (N, as NH_4_NO_3_) to maintain a N:P molar ratio of 30. Such manipulation mimics the mean values of molar TN:TP molar ratios (N:P = 30) found in Mediterranean lakes after Saharan dust-deposition inputs^50^.

### Physical variables

Daily surface-irradiance values and vertical profiles of the penetration of solar radiation in the water column were recorded using air and submersible BIC Compact 4-Channel radiometers (Biospherical Instruments Inc., CA, USA) with three channels in the UVR region (305, 320, and 380 nm) and one broad-band channel for PAR (400–700 nm). *Kd* values were determined from the slope of the linear regression of the natural logarithm of downwelling irradiance vs. depth for each wavelength considered. Continuous temperature profiles (resolution = 0.10 °C; accuracy =  ± 0.15 °C) were recorded from surface to bottom of the lakes using a multiparametric probe (Hanna HI9828-0, USA).

### Chemical variables

Samples for TP, TN and Si were placed in 300 mL PET bottles, frozen at − 20 °C, and analysed following standard protocols^48^. For DOC determinations, aliquots of 150 mL were filtered through pre-combusted Whatman GF/F filters (25 mm in diameter), placed in glass vessels, acidified with 100 µL of 1 N HCl (2% final concentration) and measured using a TOC analyser (Shimadzu, model 5000, Japan)^51^.

### Biological variables

For Chl *a*, 300-mL samples were filtered onto Whatman GF/F filters (25 mm in diameter), and stored at − 20 °C until analysis (Supplementary text S1).

Phytoplankton abundance was determined following the Utermöhl method^52^ from samples fixed with alkaline Lugol’s (~ 1% vol/vol) preserved in 125-mL brown glass bottles (Supplementary text S2).

For primary production, 50-mL samples were inoculated with labelled NaHCO_3_ (5 µCi; Perkin Elmer, Inc. USA) to measure inorganic ^14^C incorporation^53^, and incubated in situ during 4 h centred at local noon (Supplementary text S3).

### Data and statistical analyses

One-way analysis of the variance (ANOVA) was used to test significant differences among ln response ratios (lnRR; see Supplementary text S4) of the lakes tested. To test the effects of the TN:TP ratio, in situ temperature, and mean total irradiance received by communities on the P^c^ response to UVR, CO_2_, Mix, Nut, and their interaction, a five-way analysis of the covariance (ANCOVA) was performed with UVR, CO_2_, Mix, Nut and lake, as fixed factors, and TN:TP ratio, in situ temperature, and mean total irradiance, as co-variables. We considered our lakes to be a fixed factor because all communities were exposed to the same experimental manipulation. We included the above mentioned co-variables in the ANCOVA analyses because they are environmental drivers that often operate on similar time scales as P^c^ in surface waters and therefore could potentially modulate the P^c^ responses to UVR, CO_2_, Mix, and Nut and their interaction. Prior to the ANOVA and ANCOVA analysis, assumptions of normality (by Q–Q plot residual analysis and Shapiro–Wilk’s test) and homoscedasticity (by Levene’s Equal Variance test) were checked. Homogeneity of regression slopes between *Kd*_PAR_ and co-variables were checked through Pearson’s correlation analysis, and linearity between P^c^ and co-variables through dispersion plots. Differences among and within treatments and/or lakes were detected using a post hoc least-significant difference test. Finally, Student’s *t* test was used to compare global mean irradiances received by samples during exposure to static and mixing treatments.

Single and interactive UVR, CO_2_, Mix and Nut effects on P^c^ were quantified using natural logarithm response ratios (lnRR) according to the corrected formulation of Harvey et al*.*^54^ (Supplementary text S4). The relationship between the lnRR single, double, triple, and interactive drivers tested and the *Kd*_PAR_ gradient on P^c^ were assessed by polynomial regression analyses. We used non-linear regression fits because: (1) they explained a higher proportion of the total variance of the P^c^ by *Kd*_PAR_ than when using linear regression models (R^2^ < 0.40 in all interactive effects); and (2) we obtained lower values of Akaike’s information criterion (AIC) resulted in all interactions when compared with linear regression models (AIC_polynomial_ [ranging between − 5.71 and − 25.71]; AIC_linear_ [ranging between − 3.29 and − 17.26]). After regression analyses, assumption of normal distribution was checked through residual analyses.

## Supplementary information

Supplementary Information 1.

Supplementary Information 2.

## Data Availability

All data used in this study are included in the manuscript and supplementary information, and will be available under request to the corresponding author.
