# Supplementary material for: Multiple interacting environmental drivers reduce the impact of solar UVR on primary productivity in Mediterranean lakes
Source: Sci Rep. 2020 Nov 13;10:19812. doi: 10.1038/s41598-020-76237-5 (PMC7666193; doi:10.1038/s41598-020-76237-5)
Supplement: Supplementary file 2 — Supplementary Information 2. [file 41598_2020_76237_MOESM2_ESM.doc]

Multiple interacting environmental drivers reduce the impact of solar UVR on primary productivity in Mediterranean lakes

Marco J. Cabrerizo^*^, E. Walter Helbling, Virginia E. Villafañe, Juan M. Medina-Sánchez, Presentación Carrillo

*Corresponding author: Marco J. Cabrerizo email: marcojc@uvigo.es

**Supplementary text S1**

After thawing the samples, photosynthetic pigments were extracted in 90% acetone under dark conditions at 4ºC for 24 h [[^3^](#_ENREF_3)], and measured using a fluorometer (Perkin Elmer LS55, USA). A chlorophyll *a* standard from spinach (Sigma-Aldrich, USA) was used as a standard to calibrate the fluorometer.

**Supplementary text S2**

50-mL aliquots were settled in a Utermöhl chamber for 48 h to ensure complete sedimentation of the smallest algal species, counted under 400× and 1000× magnification using an inverted microscope (Carl Zeiss AX10, LCC, USA) and identified to a genus level. For each sample, 400 cells of the most abundant species were counted, and 20 cells of each species were measured to estimate biovolume [[^1^](#_ENREF_1)]. Biovolume was transformed into biomass following appropriate conversion factors of Rocha & Duncan [[^2^](#_ENREF_2)].

**Supplementary text S3**

After solar-radiation exposure, the organic carbon (C) retained in phytoplankton (i.e. primary production, PP) was determined by filtering the samples through Whatman GF/F fibreglass filters (25 mm in diameter) under low pressure (< 100 mm Hg) to avoid cell breakage and placing the filters in 20-mL vials. The C excreted by phytoplankton (EOC) was determined from 4 mL of the GF/F filtrates. Samples for PP and EOC were placed in scintillation vials, acidified with 1N HCl (2%) to remove inorganic radiocarbon, and maintained open in a hood for 24 h, as recommended by Lignell [[^4^](#_ENREF_4)]. After that, vials were filled with scintillation cocktail (Ecoscint A, USA) and incorporated C was measured using a scintillation counter equipped with autocalibration (Beckmann LS-6000 TA, USA). Total CO_2_ in water samples was calculated from alkalinity and pH measurements made in water samples using an automatic potentiometric titrator (Titrando 905, Metrohm, USA, Inc.). In all calculations, the total activity of ^14^C added was considered, and dark values were subtracted from the corresponding light values. Total primary production was calculated as the sum of PP and EOC, whereas that of the productivity rates (P^c^, h^-1^), equivalent to the intrinsic growth rates [[^5^](#_ENREF_5)], were calculated by dividing the total PP by phytoplankton C biomass.

**Supplementary text S4**

Individual, double, triple and interactive ln response ratios (lnRR) were calculated as follow:

lnRR_single_ = ln (P^c^_single_ / P^c^_control_)

lnRR_double_ = ln (P^c^_double_ / P^c^_single1_) – ln (P^c^_single2_ / P^c^_control_)

lnRR_triple_ = ln (P^c^_triple_ / P^c^_single1_) – ln (P^c^_single2_ / P^c^_control_) – ln (P^c^_single3_ / P^c^_control_)

lnRR_Interactive =_ ln (P^c^_Interactive_ / P^c^_single1_) – ln (P^c^_single2_ / P^c^_control_) – ln (P^c^_single3_ / P^c^_control_) – ln (P^c^_single4_ / P^c^_control_)

P^c^_control_ samples being exposed to -UVR, -CO_2_, Stat, and Amb. For lnRR_single_, P^c^_single_ represents the samples exposed to +UVR, -CO_2_, Stat and Amb for the UVR effect; -UVR, +CO_2_, Stat and Amb for CO_2_ effects; -UVR, -CO_2_, Fluc and Amb for Mix effect, and -UVR, -CO_2_, Stat and Enr for Nut effect. Because in our case the UVR was the main driver in terms of the effects on P^c^, we considered only 2- and 3-level interactions in which UVR was present, whereas the remaining interactions were omitted from the present study. Thus, P^c^_double_ represents samples simultaneously exposed to combinations of two of the factors tested, i.e. +UVR, +CO_2_, Stat and Amb for the UVR×CO_2_ interaction, +UVR, -CO_2_, Fluc and Amb for the UVR×Mix interaction, and +UVR, -CO_2_, Stat and Enr for the UVR×Nut interaction (see factors above). P^c^_triple_ represents samples exposed to combinations of three drivers tested, i.e. +UVR, +CO_2_, Fluc, and Amb for the UVR×CO_2_×Mix interaction; +UVR, +CO_2_, Stat, and Enr for the UVR×CO_2_×Nut interaction; and +UVR, -CO_2_, Fluc, and Enr for the UVR×Mix×Nut interaction. P^c^_Interactive_ represents samples exposed to all drivers simultaneously. For lnRR double, triple and interactive, P^c^_single1_ represents samples exposed to +UVR, -CO_2_, Stat and Amb whereas that P^c^_single2_, , P^c^_single3_ and, P^c^_single4_ represent samples exposed to the individual effect (CO_2_, Mix and Nut) of the other three drivers tested, as appropriate.

Standard deviation (*s*) for each lnRR was calculated as the pooled *s* for equal size samples [[^6^](#_ENREF_6)]:

*s* = √*s_a_*^2^ *+*… *s*_z_^2^ / *z*

*s*_a_^2^ and *s_z_*^2^ being the standard deviation obtained from three replicate of P^c^ measured from *a* to *z* treatments, respectively, and *z* the number of treatments considered in each type of interaction outlined above.

We chose lnRR to estimate the effect size over other methods because its robustness and high capacity to detect true effects in small/medium sample sizes [[^7^](#_ENREF_7)]. Also, we followed this approach to quantify the interactive effect of the drivers tested because the underlying model of such metric is multiplicative [[^8^](#_ENREF_8)], and this model is also thought to be more biologically realistic than an additive model [[^9^](#_ENREF_9)]. A value > 0 represents a net stimulatory effect, whereas a value < 0 represents an inhibitory effect.

**Supplementary figure 1.-** Productivity rates (P^c^, h^-1^) in phytoplankton communities from Lakes Río Seco Superior (RSS), Aguas Verdes (AV), Lagunillos de la Virgen (LV), Las Yeguas (LY), La Caldera (LC), Santos Morcillo (SM), San Pedro (SP), Colgada (CO) and Morenilla (MO) exposed to two radiation treatments (+UVR, > 280 nm (white bars); and -UVR, > 400 nm (black bars)), two pCO_2_ (-CO_2_, 400 ppm; and +CO_2_, 750 ppm), two mixing regimes (static vs. fluctuating) and two nutrient levels (ambient vs. enriched). The bars represent the mean of three replicates, and the vertical lines the standard deviation. Note the different y-axis scale for LC respect to the other lakes.

**Table S1.-** Mean ultraviolet (UV-B, 305 nm; UV-A, 320 and 380 nm) and photosynthetically active radiation (PAR) (in W m^-2^) received by phytoplankton communities from Lakes Río Seco Superior (RSS), Aguas Verdes (AV), Las Vírgenes (LV), Las Yeguas (LY), La Caldera (LC), Santos Morcillo (SM), San Pedro (SP), Colgada (CO) and Morenilla (MO) when incubated at a fixed depth (Static, 0.5 m) or vertically moved between water surface and 3 m depth (fluctuating) during the incubation period (4 h).

|  | Static (0.5 m) | | | | Fluctuating (0-3 m) | | | |
| --- | --- | --- | --- | --- | --- | --- | --- | --- |
| Lake | 305 nm | 320 nm | 380 nm | PAR | 305 nm | 320 nm | 380 nm | PAR |
| RSS | 0.02 | 0.13 | 0.51 | 353.51 | 0.01 | 0.06 | 0.30 | 271.49 |
| AV | 0.02 | 0.14 | 0.49 | 276.05 | 0.01 | 0.07 | 0.31 | 197.81 |
| LV | 0.01 | 0.14 | 0.51 | 298.44 | 0.01 | 0.11 | 0.43 | 253.44 |
| LY | 0.02 | 0.12 | 0.40 | 220.83 | 0.01 | 0.08 | 0.32 | 178.54 |
| LC | 0.05 | 0.27 | 1.11 | 410.89 | 0.03 | 0.18 | 0.61 | 295.07 |
| SM | 0.05 | 0.28 | 0.68 | 366.65 | 0.03 | 0.18 | 0.44 | 276.21 |
| SP | 0.04 | 0.27 | 0.68 | 371.13 | 0.03 | 0.18 | 0.45 | 279.59 |
| CO | 0.04 | 0.25 | 0.63 | 350.85 | 0.03 | 0.16 | 0.41 | 264.31 |
| MO | 0.04 | 0.24 | 0.69 | 370.37 | 0.03 | 0.16 | 0.45 | 279.01 |
| Mean | 0.87±0.28 | | | 335.41±58.90 | 0.57±0.15 | | | 255.05±39.87 |

**Table S2.-** Results of the five-way analysis of the covariance for the effects of ultraviolet radiation (UVR), carbon dioxide (CO2), fluctuating light regime (Mix), nutrients enrichment (Nut), lake, and their interaction on primary productivity. *In situ* temperature, total nitrogen:phosphorus (N:P) ratio and surface irradiances represents co-variables. df are degrees of freedom, and *F*-test are values of *F-Snedecor* test.

| Covariables | df | *F*-test | p-value |
| --- | --- | --- | --- |
| *in situ* temperature | 8 | 0.09 | 0.82 |
| Total N:P ratio | 8 | 0.08 | 0.78 |
| Surface irradiances | 8 | 0.01 | 0.95 |
| Factors | df | *F*-test | p-value |
| UVR | 1 | 1621.45 | < 0.001 |
| CO_2_ | 1 | 50.26 | < 0.001 |
| Mix | 1 | 0.09 | 0.77 |
| Nut | 1 | 2.78 | 0.06 |
| Lake | 8 | 1014.75 | < 0.001 |
| UVR×CO_2_×Mix×Nut×Lake | 8 | 0.51 | < 0.01 |

**Table S3.-** Results of the polynomial regression fit of the natural logarithm response ratios (lnRR) of ultraviolet radiation (UVR), nutrients (Nut), carbon dioxide (CO_2_), light regime (Mix) and their two-, three and four level interaction as a function of the underwater light environment (KdPAR) on primary productivity (P^c^).

| lnRR | *F*-test | R^2^ | p-value |
| --- | --- | --- | --- |
| UVR | 13.30 | 0.82 | < 0.01 |
| Nut | 21.14 | 0.88 | < 0.01 |
| CO_2_ | 1.02 | 0.10 | 0.42 |
| Mix | 0.31 | 0.20 | 0.75 |
| UVR×Nut | 6.35 | 0.68 | < 0.05 |
| UVR×CO_2_×Mix | 6.58 | 0.69 | < 0.05 |
| UVR×CO_2_×Nut | 7.45 | 0.71 | < 0.05 |
| UVR×Mix×Nut | 57.90 | 0.95 | < 0.001 |
| Interactive | 14.58 | 0.83 | < 0.01 |

**Supplementary references**
